# The inhibitory effect of agricultural fiscal expenditure on agricultural green total factor productivity

**DOI:** 10.1038/s41598-022-24225-2

**Published:** 2022-12-03

**Authors:** Shuguang Wang, Jiaying Zhu, Lang Wang, Shen Zhong

**Affiliations:** 1 Harbin University of Commerce, Harbin, Heilongjiang China; 2 School of Finance, Dongbei University of Finance and Economics, Dalian, Liaoning China

**Keywords:** Environmental sciences, Environmental social sciences

## Abstract

Sustainable development of agriculture is the basis for achieving social sustainable development. As the basic industry of national economy, green development of agriculture has become an important support for building an environment-friendly society. Agricultural fiscal expenditure is a direct channel for the government to support agriculture and promote agricultural transformation. It is important to analyze the impact of agricultural fiscal expenditure (AFE) on agricultural green total factor productivity (AGTFP) for sustainable agricultural development. Therefore, this paper employs the random effect model and spatial Durbin model to empirically analyze the direct effect and spatial spillover effect of AFE on AGTFP by using the agricultural panel data of 30 provinces in China from 2008 to 2020. Then, by taking the policy proposal as the time node, this paper also conducts a time heterogeneity analysis to measure the impact of policy enactment on AFE and AGTFP. The main conclusions are as follows: (1) AGTFP exists significant positive spatial spillover effect. The "radiation effect" of agricultural green development is significant. (2) AFE can significantly reduce the AGTFP in the local area, that is, 1% increase of AFE in the local area will reduce AGTFP by 0.037%. At present, agriculture is still yield-oriented. The improvement of AFE in the local area will lead to the expansion of local agricultural production and increase pollution emission. (3) AFE has a significant negative spatial spillover effect on AGTFP, that is, for every 1% increase in AFE, the AGTFP will decrease 0.123% in geographically similar areas, while the AGTFP will decrease by 0.116% in economically and geographically similar areas. It is obvious that AFE will promote the optimization of agricultural production conditions in the province, with the "demonstration effect" on the surrounding areas, the enthusiasm of production in the surrounding areas will increase, thus expanding the pollution emission. (4) According to the analysis of different periods, AFE has a negative impact on AGTFP mainly before the reform innovation is proposed in 2015. It indicates that reform policies have a significant impact on agricultural sustainability.

## Introduction

As the basic material production sector and the foundation of the national economy, the sustainable development of agriculture is particularly important for the green development of the national economy. Obviously, China's agricultural economy has made great achievements, but it has also brought about serious environmental problems^1^. The excessive use of agricultural inputs, such as fertilizers and pesticides, or the improper disposal of agricultural wastes such as livestock and poultry manure, crop straw and farm residue film will increase agricultural pollution emissions^2,3^. According to *China's Ecological Environment Statistical Annual report in 2020*, the national chemical oxygen demand emissions from wastewater were 25.648 million tons, it should note that agriculture discharged 15.932 million tons; the national ammonia nitrogen emissions were 984,000 tons, with agriculture accounting for 254,000 tons. At present, agriculture has surpassed industry as the largest non-point source pollution industry in China, it not only seriously endangers the sustainable development of agriculture, but also directly threatens the ecological security, agricultural product safety and human health in China^4^. Therefore, it is an urgent task to improve the level of green agriculture while maintaining the development of agricultural economy, and promote the sustainable development of agriculture. It is also the requirement for China to develop biological agriculture and promote the development of circular bioeconomy.

Among the many factors affecting the sustainable development of agriculture, it is generally agreed that government macro-control is one of the effective means to guide cleaner agricultural production, which is the key to the successful implementation of cleaner production. The AFE refers to various funds used for agriculture in the national financial expenditure, mainly including agricultural capital construction expenditure, agricultural science and technology expenses, agricultural subsidies and so on. It is an important part of government macro-control, providing public goods for agricultural production, enhancing the motivation of agricultural producers, and supporting and protecting the sustainable development of agriculture^5^. As a direct channel for the government to support agriculture, on the one hand, AFE can improve the content of agricultural science and technology and increase the potential for sustainable agricultural development^6^. On the other hand, the improvement of agricultural production conditions influenced by yield orientation may make operators blindly expand their scale and increase agricultural pollutant emissions^7^. In summary, analyzing the impact of AFE on sustainable agricultural development is important to improve government allocation efficiency and promote green agricultural development.

## Literature review

As the primary industry, agriculture supports the development and progress of national economy. The green development of agriculture is the foundation of sustainable economic development. Meanwhile, compared with bio-manufacturing and bio-energy^8,9^, agriculture has the characteristics of development globalization. It is the only way to improve the agricultural system to transform crop varieties and improve the performance of agricultural products through biotechnology. The analysis of agricultural green efficiency is conducive to promoting the development of biological agriculture, thus promoting the development of China's circular bioeconomy^10^. Zhang et al.^11^ proposed that there are three main aspects that affect green TFP: technology, economy and government. Therefore, numerous studies on the influencing factors of AGTFP have been carried out accordingly. Wu and Zhang^12^ divided Internet technology into technology Internet and platform Internet, and analyzed its impact on forestry green total factor productivity. The conclusion shows that both the Internet of science and technology and the Internet of platform have a positive impact on the clean production of forestry in the short term. In the long term, the technology Internet hinders the transformation of green technology efficiency and green technology progress, while the platform Internet still has a positive impact on both. Yu et al.^13^ proposed that the implementation of carbon trade pilot policies has a significant promoting effect on agricultural green total factor productivity. Liu and Lv^14^ verified the nonlinear relationship between rural human capital and AGTFP, and they found there is a significant double-threshold effect between rural human capital and AGTFP at different levels of agricultural physical capital and agricultural economic development. Fang et al.^15^ believed that the government's agricultural subsidy system can promote the green agricultural development. In addition, crop insurance, as an effective risk protection mechanism, can significantly increase agricultural green total factor productivity, many scholars, such as Li et al.^16^, Huang et al.^17^ and He et al.^18^ carried out analysis based on this.

AFE is a direct channel of government support for agriculture, including agriculture, forestry, water conservancy, south-to-north water transfer, poverty alleviation, comprehensive agricultural development, and other agricultural water affairs. It can influence the economic and environmental benefits of agriculture through farmland and water infrastructure construction, scientific and technological inputs, and agricultural support subsidies. Most of the existing studies have focused on analyzing the impact of AFE on the economic efficiency of agriculture. Zeng et al.^19^ used a vector autoregressive (VAR) model and found that agricultural fiscal spending is much more effective in reducing rural poverty than agricultural development. Iganiga and Unemhili^20^ found that federal government agricultural spending is positively associated with agricultural output, but with a 1-year lag. Chandio et al.^21^ argued that there is a long-run relationship between government agricultural expenditure, agricultural output and economic growth in Pakistan. Agricultural output, government expenditure has significant impact on economic growth in Pakistan. Anderu and Omotay^22^ found that disruption of government spending in the agricultural sector adversely affects the growth of agricultural output in Nigeria. Moreover, Ewubare and Eyitope^23^, Guo et al.^24^, Xing^25^ and others who come to similar conclusions that government spending on agriculture is beneficial to agriculture to expand production, increase output and facilitate economic efficiency. In contrast, fewer studies have been conducted on the impact of AFE on agricultural environmental efficiency. Only a few studies have discussed the AFE in the context of drivers of green efficiency in agriculture^26,27^. There is no specific literature to analyze the specific transmission mechanism of AFE on agricultural green efficiency.

To sum up, although many scholars have extensively studied the impact of AFE on agricultural development and investigated the effect of AFE on agricultural production, the following aspects still need to be improved: (1) Most of the analyses in the literature focus on the economic benefits, while the environmental benefits of AFE on agricultural production are always neglected. (2) Due to the "cohort effect" of the government and the characteristics of agricultural production, there is a strong spatial correlation between AFE and AGTFP. However, there is a gap in the research on the spatial spillover effects of AFE and AGTFP.

The possible innovations of this paper are: (1) In terms of research perspective, for the first tie, this paper takes the impact of AFE on agricultural environmental benefits as an entry point. It incorporates undesired outputs in the analysis of its impact on agricultural production and measures its impact on sustainable development. (2) In terms of research methodology, the spatial econometric model is applied for the first time. The spatial geographical distance matrix and the nested economic geographical distance matrix are used to examine the spatial correlation and spatial spillover effects of AFE and AGTFP, respectively. Meanwhile, the instrumental variable method is used to control the endogeneity problem. (3) In terms of sample selection, the panel data of 30 China’s provinces from 2007 to 2020 are used as the sample for the first time. The impact of AFE on agricultural green development is analyzed from both theoretical and empirical aspects.

## Research hypothesis

**Figure 1:**
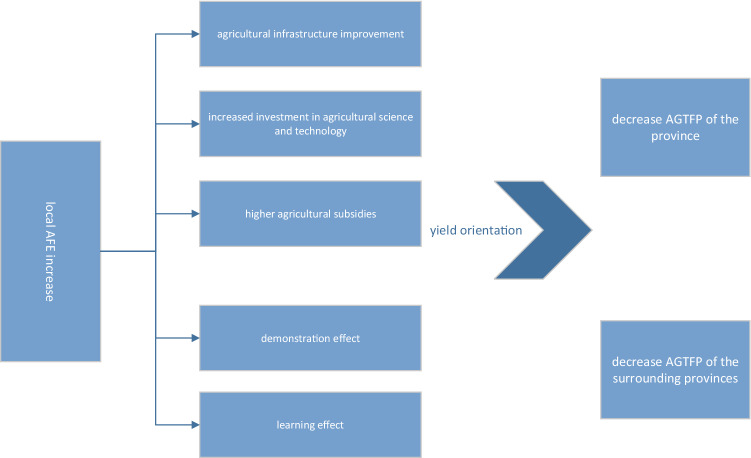
The transmission mechanism.

AFE is an important channel for the government to support and protect agricultural development. To further analyze its effect on AGTFP, based on existing theoretical and empirical studies, this paper draws Fig. 1 to describe the mechanism of AFE's influence on AGTFP. And the following two hypotheses are proposed.

Firstly, AFE is mainly used for investment in agricultural infrastructure development, agricultural subsidies, and science innovation funds. First of all, agricultural infrastructure construction can significantly improve the efficiency of agricultural public goods supply^28^. A large number of agricultural public goods and public services are needed in agricultural production and operation. For example, rural roads, water and electricity facilities, drainage and irrigation systems, public information services, and so on. The effective supply of these public goods and services facilitates agricultural producers in the region to expand their production. Secondly, the funding for science and technology innovation in AFE is conducive to improving the quality of agricultural production input factors, such as agricultural machinery and fertilizers, and improving the content of agricultural science and technology is conducive to the transformation of green modernization of agriculture^29^. However, it is worth noting that transforming development through innovation requires long-term science and technology investment. It is difficult to enhance AGTFP with short-term investment. Finally, agricultural support subsidies can correct externalities of agricultural production^30^. Agricultural production has positive externalities, and its social benefits are greater than private benefits. When agricultural producers produce according to private marginal costs and benefits, the actual production is smaller than the socially optimal production. The financial subsidies included in AFE can correct the externality of agricultural production and improve the enthusiasm of agricultural producers. However, the current production orientation relies mainly on yield orientation rather than technology orientation, thus expanding the current crude scale of operation and reducing the AGTFP in the region.

In summary, hypothesis 1 is proposed: AFE has a significant inhibitory effect on AGTFP, the growth of local AFE will decrease the AGTFP in the local area.

Secondly, the interaction of spatial data produces spatial dependence^31^. Anselin and Griffith^32^ proposes that almost all spatial data are spatially dependent. As an important component of government macro-control, understanding the spatial spillover effect of AFE on AGTFP is of great significance to the sustainable development of agriculture. On the one hand, the increase of AFE in neighboring provinces will lead to the improvement of neighboring agricultural infrastructure, the increase of scientific and technological investment, as well as the increase of agricultural subsidies. As agricultural productivity improves, the production potential increases, thus generating the "learning effect" in the region, that is, agricultural producers in the province will imitate and learn in order to improve agricultural production capacity and expand efficiency^33^. On the other hand, the increase of AFE in neighboring provinces improves agricultural production conditions. Due to the "demonstration effect" of the provincial governments in the surrounding area^34^, agricultural producers in the region perceive that they will also receive financial support in the future. Therefore, with the improvement of agricultural production enthusiasm, agricultural producers rely on output guidance to expand profits and increase production, thus further reducing AGTFP in the local area.

In summary, hypothesis 2 is proposed: AFE has a negative spatial spillover effect, and the growth of AFE in neighboring provinces will significantly reduce the level of AGTFP in the local area.

## Method and model

### SBM-Malmquist–Luenberger Productivity Index

Faced with the effectiveness of resources and the increasingly serious problem of severe pollution, the significance of green economy and sustainable development has become more and more important. Resource and environment problems are no longer endogenous variables affecting agricultural development. They have become more rigid constraints limiting the quality of agricultural development. Compared with the traditional total factor productivity (TFP) that considers single desired output, the efficiency evaluation of green total factor productivity (GTFP) which includes undesired output index is more comprehensive and objective. In this paper, AGTFP is defined as the input–output efficiency of agricultural factors under environmental constraints. Firstly, the SBM model is used to construct the optimal technology frontier and the efficiency evaluation of each decision-making unit relative to the reference technology. On this basis, the Malmquist–Luenberger productivity index is used to measure the dynamic trend of AGTFP and its decomposition.

The SBM model considers the problem of neglecting slack variables in traditional DEA models and proposes the SBM-Malmquist index based on a non-radial perspective. The efficiency values of the model will be more precise as the slackness of input and output indicators changes. Since the operational production of agriculture is inevitably accompanied by various pollutants and wastes such as chemical oxygen demand, total nitrogen, total phosphorus, and ammonia nitrogen, referring to Hong and Shi^35^, Tone and Sahoo^36^, this paper incorporates undesirable outputs into the model. Suppose that there exist $n$ decision units (DMU_S_), input indicator $x\in {T}_{u}$, desirable output $y\in {T}_{s}$, undesirable output $b\in {T}_{r}$, The matrix can be expressed as follows:

$$X=\left\{{x}^{1},\dots ,{x}^{n}\right\}\in {T}_{u\times n}$$

$$Y=\left\{{y}_{1},\dots ,{y}_{n}\right\}\in {T}_{s\times n}$$

$$b=\left\{{b}_{1},\dots ,{b}_{n}\right\}\in {T}_{r\times n}$$

$$X>0,Y>0,b>0$$

Under the weak disposability assumption, with constant returns to scale, the set of production possibilities can be defined as:

1 $$\left\{\left(x,y,b\right)x\ge X\lambda ,y\ge Y\lambda ,b\ge B\lambda ,\lambda >0\right\}$$

The SBM model including the undesirable output is set as follows:

2 $$\begin{aligned} & E_{0} = min\frac{{1 - \frac{1}{U}\sum\nolimits_{{i = 1}}^{U} {\frac{{s_{{i0}}^{x} }}{{x_{{i0}} }}} }}{{1 + \frac{1}{{J + R}}\left( {\sum\nolimits_{{j = 1}}^{J} {\frac{{s_{{j0}}^{b} }}{{y_{{j0}} }}} + \sum\nolimits_{{r = 1}}^{R} {\frac{{s_{{r0}}^{y} }}{{y_{{r0}} }}} } \right)}} \\ & s.t.\quad x_{0} = X\lambda + s^{x} \\ & y_{0} = Y\lambda - s^{y} \\ & b_{0} = B\lambda + s^{b} \\ s^{x} ,s^{y} ,s^{b} ,\lambda \ge 0 \\ \end{aligned}$$

where, $$i,j,r$$ respectively represent the number of input variables, the number of undesired output variables and the number of desired output variables; $${s}_{i}^{x},{s}_{r}^{y},$$ $${s}_{j}^{b}$$ are the input slack vectors, desired output and undesired output slack vectors. $$\lambda$$ is the weight variable, $$X\lambda$$ is the optimal combination of production inputs, $$Y\lambda$$ is the optimal combination of desired outputs, $$B\lambda$$ is the optimal combination of undesired outputs. And $$(X\lambda ,Y\lambda ,B\lambda )$$ is the "virtual optimal production situation", which is a linear mixture of all the actual production situations. The implication of Eq. (2) is that if the actual decision-making unit wants to increase the effectiveness, it must reduce the input and the undesired output and increase the desired output. $${E}_{0}$$ is the SBM model constructed to express the input–output efficiency of ($${x}_{0}, {y}_{0},{b}_{0})$$. The purpose of taking the minimum value is to make the efficiency value as small as possible, that is, the improvement space as large as possible. It can be regarded as a requirement for the improvement of current production efficiency.

The Malmquist–Luenberger productivity index model, with reference to time periods f and f + 1, measures the DMU’s input–output efficiency by calculating the ratio of the distance function, and it is used to evaluate the output efficiency of the DMU in different periods. According to the Global Malmquist index, assuming that the period function is z, the $f$-th period production possibility set is ${s}_{f}=\left\{\left({y}_{f},{b}_{f}\right):X\;can\;produce\;Y\;and\;B\right\}$, and the global production possibility set is ${s}_{g}={s}_{1}\cup {s}_{2}\cup \dots \cup {s}_{z}$. ${E}_{g}({x}_{f},{y}_{f},{b}_{f})$ represents the efficiency value of the $f$-th period when referring to the global production possibility set. The global Malmquist – Luenberger index is expressed as:

3 $$\begin{aligned} ML\left( {x^{{f + 1}} ,y^{{f + 1}} ,b^{{f + 1}} ,x^{f} ,y^{f} ,b^{f} } \right) & = \frac{{E^{g} \left( {x^{{f + 1}} ,y^{{f + 1}} ,b^{{f + 1}} } \right)}}{{E^{g} \left( {x^{f} ,y^{f} ,b^{f} } \right)}} \\ & = \frac{{E^{{f + 1}} \left( {x^{{f + 1}} ,y^{{f + 1}} ,b^{{f + 1}} } \right)}}{{E^{f} \left( {x^{f} ,y^{f} ,b^{f} } \right)}} \times \left( {\frac{{E^{g} \left( {x^{{f + 1}} ,y^{{f + 1}} ,b^{{f + 1}} } \right)}}{{E^{{f + 1}} \left( {x^{{f + 1}} ,y^{{f + 1}} ,b^{{f + 1}} } \right)}} \times \frac{{E^{f} \left( {x^{f} ,y^{f} ,b^{f} } \right)}}{{E^{g} \left( {x^{f} ,y^{f} ,b^{f} } \right)}}} \right) \\ & = TEC \times TC \\ \end{aligned}$$

Among them, when the ML value is greater than 1, it indicates that the AGTFP has an increasing trend from period f to period f + 1; when the ML value is less than 1, it indicates that the AGTFP has a decreasing trend from period f to period f + 1, and the efficiency has decreased. In addition, TEC (technical efficiency change index) is used to measure the management level of the DMU; TC (technical progress index) is used to measure the technical progress of the DMU. If $TEC/TC>1,$ it means that the technical efficiency/technical progress of the sample has increased relative to the previous period. Conversely, it indicates that the technical efficiency/technical progress of the measured sample is unchanged or decreased relative to the previous period.

### Spatial correlation test

The Moran index is able to analyze whether the variables are spatially autocorrelated^37,38^. The global Moran index gives the value of correlation for all data (Eq. 4), and the local Moran index is used to analyze single element correlation (Eq. 5).

4 $${Mora{n}^{^{\prime}}s I}_{iu}=\frac{n}{\sum_{i=1}^{n}\sum_{u=1}^{n}{w}_{iu}}\times \frac{\sum_{i=1}^{n}\sum_{u=1}^{n}{w}_{iu\times }({Y}_{it}-\overline{Y })({Y}_{ut}-\overline{Y })}{\sum_{i=1}^{n}({Y}_{it}-\overline{Y })}$$

5 $${Mora{n}^{^{\prime}}s I}_{it}=\frac{n\times ({Y}_{it}-\overline{Y })\times \sum_{u=1}^{n}{W}_{iu}\times ({Y}_{ut}-\overline{Y })}{\sum_{i=1}^{n}{({Y}_{i}-\overline{Y })}^{2}}$$

If $Mora{n}^{^{\prime}}s\;I>0$, it indicates the existence of spatial positive correlation; if $Mora{n}^{^{\prime}}s\;I<0$, it indicates the existence of spatial negative correlation; if $Mora{n}^{^{\prime}}s\;I=0$, the variables are spatially random.

### Spatial econometric model

The spatial econometric model is obtained by extending the traditional panel model to incorporate spatial interaction effects^39^. Agricultural production operations are inherently spatial in nature, and agricultural non-point source pollution is strongly influenced by spatial factors^5^. To further consider the influence of AFE on AGTFP, this paper constructs the spatial Durbin model (SDM). SDM model is a combination of spatial lag model (SAR) and spatial error model (SEM), which can combine the advantages of these two models and specifically analyze the direct and indirect effects of AFE on AFTFP. The model assumes that the dependent variable is influenced by the independent variables of neighboring regions in addition to the independent variables of the local region, that is, the spatial lagged value of the independent variable is added to the model. The model can reflect the spatial autocorrelation of the dependent variable, the correlation of the dependent variable with the independent variable and the correlation of the dependent variable with the independent variable of the neighboring region. It can specifically analyze the direct and indirect effects of AFE affecting AGTFP. The basic model setup is as follows:

6 $$\mathrm{y}=\delta W\mathrm{y}+\mu WX+\theta X+\varepsilon$$

where, $W$ is the spatial weight matrix, $y$ is the dependent variable,$X$ is the independent variable,$\varepsilon$ is the disturbance term, and, $\delta ,\mu ,\theta$ are the corresponding coefficients.

Based on the previous hypothesis, this paper adopts ${AGTFP}_{it}$ as the agricultural green total factor productivity indicator in the $t$-th year of the $i$-th province. AFE as the core explanatory variable, and it also incorporates the control variable C to measure the influence of AFE on AGTFP, and the final model is established as follows:

7 $$\mathrm{SDM}: {AGTFP}_{it}=\delta \sum {W}_{ij}\times {AGTFP}_{it}+\mu {W}_{ij}\times {AFE}_{it}+\theta {AFE}_{it}+{\phi }_{1}{W}_{ij}\times {C}_{it}+{\phi }_{2}{C}_{it}+{\varepsilon }_{it}$$

where, $\delta$ reflects the spatial spillover of AGTFP to neighboring regions, and the significance of this coefficient can reflect whether the dependent variable has a spatial effect. $\mu$ represents the influence of AFE in the surrounding area on AGTFP in the local area. $\theta$ reflects the influence of AFE in the local area on AGTFP in the local area, that is, the direct effect of AFE on the dependent variable. ${\phi }_{1},{\phi }_{2}$ are the coefficients of the control variables, and $\varepsilon$ is the disturbance term.

### Spatial weight matrix

Spatial weight matrices can reflect the dependencies of individuals in space, as the spatial interaction effect is widely known, the settings of spatial weight matrices are becoming more and more diverse^40^. This paper refers to previous studies^41^, and sets two weight matrices W1 and W2. Geographic distance matrix (W1): The geographic weight matrix is a measure of the importance of the relationship between two places by the distance of different points in the coordinate system. When the distance between two places is farther away, it is considered to have a lower weight. On the contrary, it is higher. The spherical distance (dis) between each point is calculated by using the latitude and longitude of the provincial capital, and its reciprocal is taken as the geographic distance matrix. Economic geographic distance matrix (W2): The degree of spatial dependence is influenced not only by geographical factors but also by economic level. Regions with similar economic levels tend to have greater spatial correlation as well. Therefore, this paper takes into account the influence of economic level, on the basis of geographic factors, to set the economic geographic distance matrix (W2). Moreover, it uses the GDP per capita from 2008 to 2020 to represent the economic level of each province, and then adopts the inverse of the absolute value of the difference to represent the economic distance between two provinces. The economic distance matrix and the geographic distance matrix are assigned a value of 0.5 respectively, which is denoted as economic geographic matrix. The spatial dependence of the variables was analyzed from both geographic and economic perspectives.

$$WG = \frac{1}{{\left| {gdp_{i} - gdp_{j} } \right|}}\quad W2 = 0.5 \times W1 + 0.5 \times WG$$

## Variable selection and data resource

### Variable selection

#### Input variable

According to the factor theory of agricultural production^42^ and the actual situation of agricultural production in China, this paper selects five input variables. The number of employees in the primary sector is used to represent labor input; the total sown area of crops as land input; the effective irrigated area as irrigation input; the fertilizer application amount as fertilizer input; and the total power of agricultural machinery as machinery input.

#### Output variable

This paper incorporates environmental factors into the traditional total factor productivity in agriculture, and divides the output variables into desirable output and undesirable output^43^. Among them, the total output value of agriculture, forestry, animal husbandry and fishery can reflect the value created by agricultural production in that year, and this indicator can depict the development of agriculture. Therefore, the total output value of agriculture, forestry, animal husbandry and fishery industry are used to represent the desirable output. Meanwhile, in order to further analyze the efficiency of green development, environmental factors are included in the input–output model. Some scholars use environmental factors as the undesirable output variable, indicating that the agricultural production process produces both the desirable output, such as product yields, and the undesirable output, such as non-point source pollution. This approach is consistent with the actual agricultural production process. Therefore, this paper includes agricultural non-point source pollution as the undesirable output indicator. It includes chemical oxygen demand, total nitrogen, total phosphorus, and ammonia nitrogen, which is one of the main agricultural production pollutions. The entropy method is used to calculate the comprehensive agricultural pollution index as the undesirable output, and the calculation process is shown in Supplementary Table S1.

#### Dependent variable

The analysis of green total factor productivity considering environmental constraints has become a research hotspot at present^44^. To further build the framework of green production analysis in agriculture, this paper selects five agricultural production input variables, then it takes agricultural non-point source pollution as the undesirable output, as well as the total output value of agriculture, forestry, animal husbandry and fishery as the desirable output. The SBM-Malmquist index is used to measure agricultural green total factor productivity (AGTFP) as the dependent variable.

#### Core explanatory variable

AFE is a direct channel for the government to support agriculture. AFE can influence the green development of agriculture by building agricultural infrastructure, promoting agricultural science and technology innovation, and increasing agricultural subsidies. On one hand, the increase of AFE can improve the green transformation of agriculture by improving agricultural production conditions, increasing the content of agricultural science and technology, and enhancing farmers' motivation^45^. On the other hand, when the agricultural transformation is incomplete and the agricultural development model is at the low end, the increase of AFE will cause agricultural producers to blindly expand their crude production patterns and reduce the green development of regional agriculture. Therefore, this paper chooses AFE as the core explanatory variable to specifically analyze the effect of AFE on agricultural green production efficiency.

#### Control variable

Based on the characteristics of agricultural development and the current production situation, in order to prevent errors in omitted variables, this paper selects the following five control variables.

1. Education level (Edu): the increase in the education level of farmers is conducive to the optimal allocation of agricultural production resources and technical efficiency^46^. At the same time, agricultural production relies more on the improvement of labor quality and technological progress, thus driving agricultural green production and changing the crude production method to improve the AGTFP. This paper adopts the education expenditure to GDP ratio to represent the education level.
2. Income disparity (Indi): There is a "resource transfer effect" of urban–rural income disparity on the rural economy. The widening of the income gap between urban and rural residents is not conducive to the stable development of the rural economy^47^. It has been shown that the widening of the urban–rural income gap inhibits the improvement of total factor productivity in agriculture. For the green development of agriculture, on the one hand, the widening income gap will reduce farmers' production motivation, lower the proportion of primary industry, thus reducing the AGTFP. On the other hand, some agricultural producers will enhance their income, strengthen technological investment, and change the low-end development mode in order to improve AGTFP. This paper uses the ratio of average annual disposable income per person in urban households to average annual net income per person in rural households to express the income gap.
3. Technology level (Tech): The improvement of technology level is conducive to breeding and screening higher quality varieties, improving agricultural production equipment, and increasing land productivity^48^. It can improve the quality of agricultural production. At the same time, it is conducive to the development of more efficient and environmentally friendly fertilizers and pesticides to reduce agricultural surface pollution. In terms of pollution treatment, the improvement of technology level is conducive to the development of pollution treatment equipment and the reduction of pollution emissions. Furthermore, the improvement of technology level can also facilitate the development of environment-friendly agriculture by expanding production scale through resource saving and yield improvement. This paper uses the ratio of science and technology expenditure to GDP to represent this variable.
4. Natural disasters (Dis): Agriculture is highly affected by natural disasters, which can directly affect agricultural production efficiency and agricultural returns^49^. The frequent occurrence of natural disasters in a region can constrain agricultural development in the current period, often resulting in a weak agricultural base in the region and making it difficult to progress in the long term. At the same time, it reduces the incentive of agricultural producers to produce, thus constraining the green transformation of agriculture in the region. Natural disasters affecting agriculture in China are mainly meteorological disasters, especially flood and drought disasters. In this paper, the proportion of areas affected by natural disasters to areas covered by natural disasters is used to represent the indicator of natural disasters. The disaster-affected area refers to the crop sown area that is reduced by more than 10% due to the disaster, and the disaster-covered area refers to the crop sown area that is reduced by more than 30% due to the disaster. It can reflect the adverse effects of flood and drought disaster on Chinese agriculture.
5. Agricultural scale (Agri): Scale operation can promote the green development of agriculture^50^. Obviously, the expansion of agricultural scale can improve the efficiency of agricultural machinery, fertilizer and agricultural chemicals, and reduce the use demand of chemical fertilizer in agriculture. The scale of agriculture is also the focus of the transformation from crude to intensive agriculture, which is conducive to the extension of the industrial chain and the development of environmentally friendly agriculture. The proportion of primary industry is used to represent the variable.

#### Data source

The panel data of 30 provinces in China are selected from the *China Statistical Yearbook* (2007–2020), the *China Stock Market Accounting Research Database* and the statistical yearbooks of each province. To maintain data stationarity, each variable is multiplied by 10 and takes logarithm. This method will not change the nature and correlation of data, and it reduces the scale of variables and increases the stationarity of data. At the same time, this paper carries out the stationarity test, and the conclusion further verifies the stationarity of the variables. The specific results are shown in Supplementary Table S2. And the descriptive statistics of the variables are shown in Table 1.

**Table 1:** Descriptive statistics of variables.

Variable	Obs	Mean	Std. Dev	Min	Max
AGTFP	390	2.371	0.080	2.181	3.182
AFE	390	− 1.435	0.630	− 2.950	0.089
Edu	390	− 1.021	0.341	− 1.725	− 0.097
Indi	390	3.335	0.175	3.012	3.754
Tech	390	− 3.296	0.512	− 4.303	1.944
Dis	390	6.072	0.289	4.998	6.702
Agri	390	0.471	0.456	− 0.790	1.745

### Spatial and temporal characteristics of AGTFP

**Figure 2:**
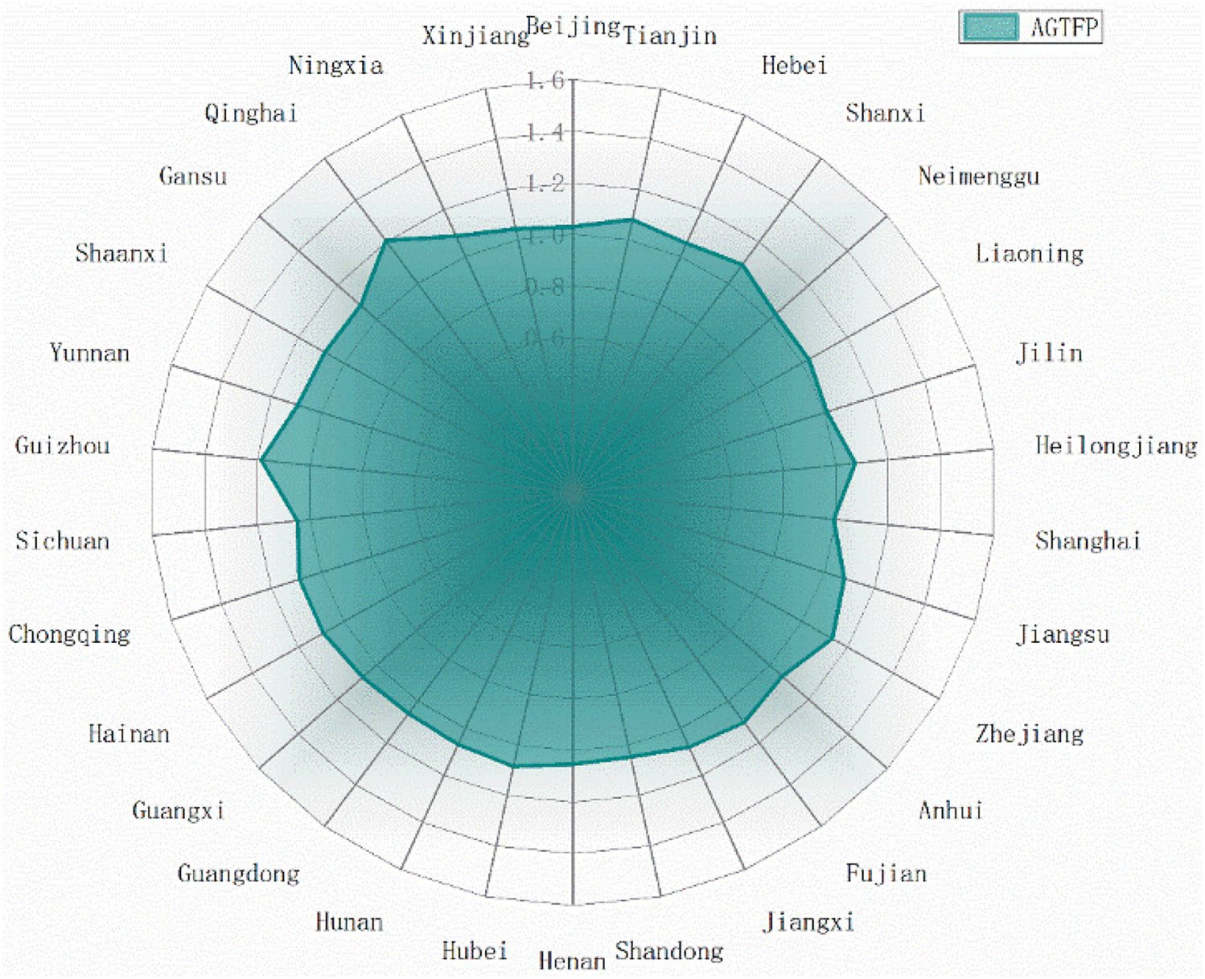
Spatial heterogeneity of AGTFP in 30 provinces.

**Figure 3:**
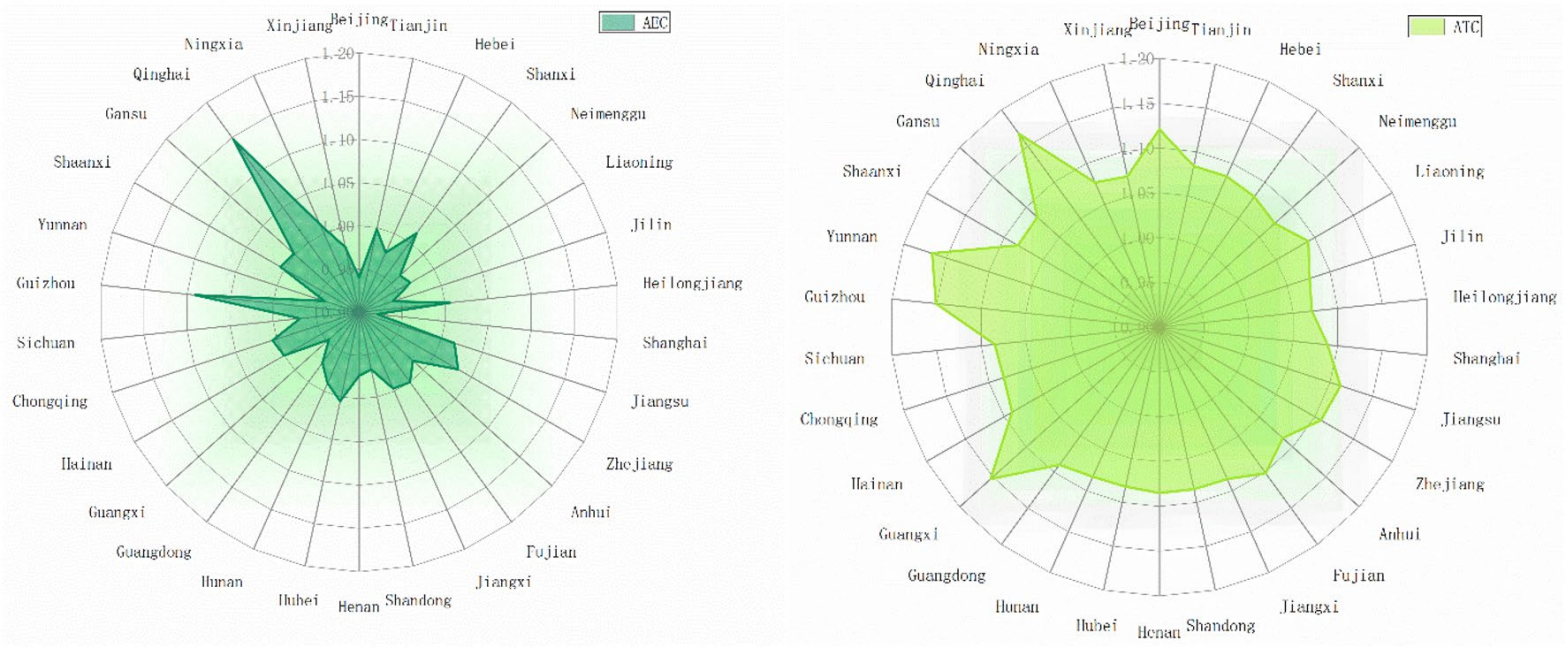
Spatial heterogeneity of AEC and ATC in 30 provinces.

Figures 2 and 3 reflect the mean values of AGTFP and its decomposition factors ATC and AEC in 30 provinces over 13 years respectively. It can be seen that the AGTFP is basically improved in the sample period of 30 provinces, and the AGTFP of 29 provinces is greater than 1. Only Shanghai has the mean value of AGTFP of 0.991. Combining the decomposition factors, it finds that Shanghai AEC is the lowest in 30 provinces, only 0.921. The regression of management level reduces the level of AGTFP. Shanghai is an international economic, financial, trade, shipping, and technology center. As of 2021, the GDP of Shanghai accounts for 3.78% of the country. The economic prosperity has caused the proportion of primary industry to be continuously compressed, and the arable land area is decreasing. Currently, Shanghai mainly focuses on leisure agriculture, tourism agriculture, and urban agriculture, and the technology level is constantly improving. However, the management level is difficult to be taken into account, and the reduced arable land area makes it more difficult to achieve intensive production. Figure 3 shows that all provinces in China have achieved technological progress, and all 30 provinces have ATC values greater than 1.06, while efficiency progress has always been neglected. It should note that only 11 provinces achieved efficiency progress during the sample period, indicating that the insufficient level of agricultural resource allocation is a great problem in the agriculture of China. The improvement of AGTFP mainly relies on technological progress, and the modernization of agriculture has greatly enhanced the development of agricultural science and technology. In recent years, there has been a trend to rely on scientific and technological innovation to drive the development of traditional agriculture. However, attention should be paid to the coordination between management level and technological progress to avoid the situation that the development of AGTFP is constrained by insufficient AEC.

**Figure 4:**
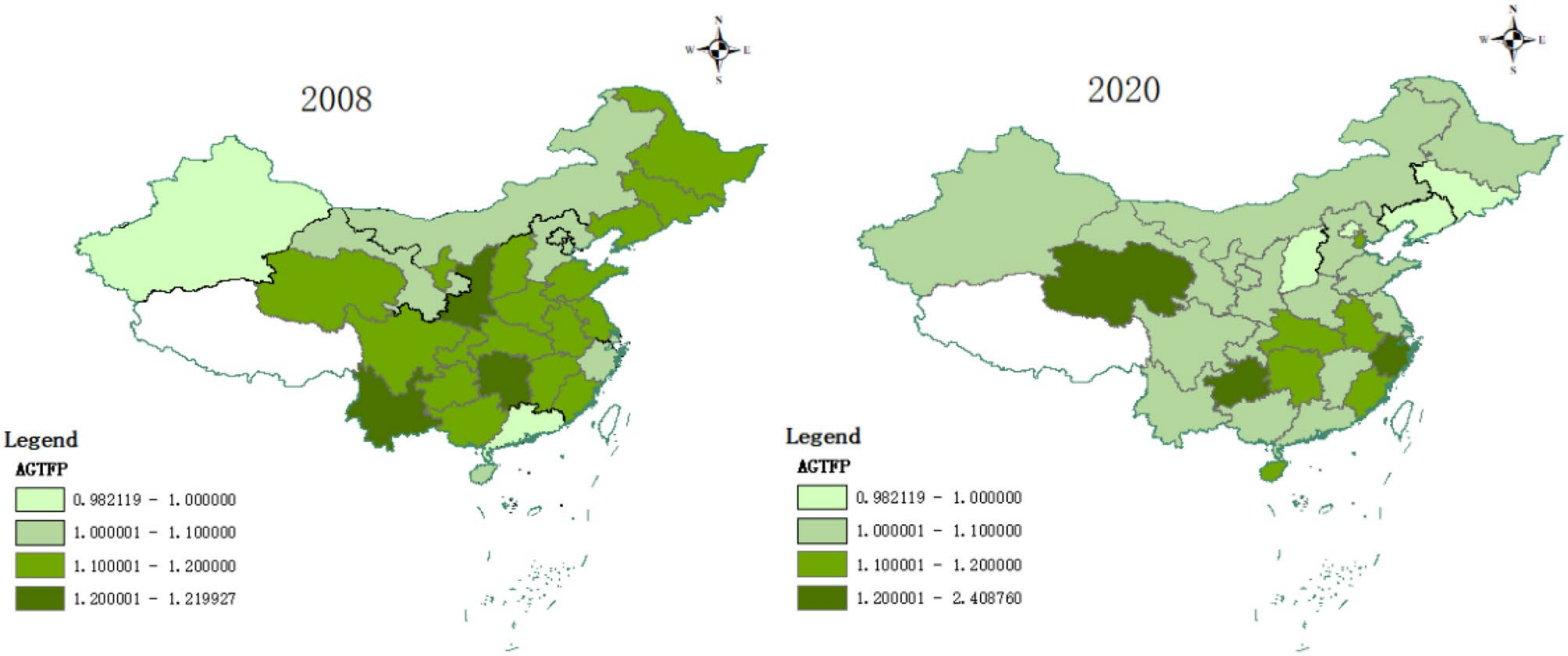
Spatial and temporal characteristics of AGTFP in 30 provinces.

The AGTFP of each province in 2008 and 2020 can be seen in Fig. 4. It reflects the heterogeneity and temporal trend of agricultural green development in each province during 13 years. Spatially, the rate of AGTFP improvement in 30 provinces slows down. The number of medium–high speed development zones has changed from 20 to 9, in line with the requirements of stable and solid green transformation of agriculture. In time, the average value of AGTFP in 30 provinces of China has increased after 13 years, rose from 1.1125 in 2008 to 1.1285 in 2020, and the development trend of agricultural green development is positive. Combining the time characteristics, the following conclusions can be drawn: firstly, some coastal areas in the southeast are always leading the country in agricultural green development. The potential reason is the high level of economic development in this region, at the same time, it has regional advantages and high-quality labor capital. It is convenient for technological innovation and learning advanced agricultural technologies from abroad. As a result, the region's AGTFP has always remained at a high level. Second, the northwest region also performs better in further improving AGTFP, such as the Xinjiang Uyghur Autonomous Region and Qinghai Province. Xinjiang Uyghur Autonomous Region regressed its agricultural green development in 2008, while achieving AGTFP growth in 2020. Qinghai Province consistently maintains a high level of agricultural green development and leaps to become a leading region in the country by 2020. Northwest China, influenced by topography and climate, human resources and transportation, has continued to improve the quality of agricultural development and develop special agriculture. In Xinjiang, for example, cotton cultivation is the pillar industry of Xinjiang agriculture. The region continues to increase the introduction and promotion of mechanized cotton harvesting and other technologies to guide the concentration of cotton cultivation in advantageous areas. Through the development of large-scale and intensive agriculture, the level of AGTFP in the region has been improved. Finally, there is degradation of agricultural green development in the Northeast. In particular, Jilin Province, Liaoning Province AGTFP has dropped significantly. The main reason is that some provinces in the northeast face depletion of agricultural resources and decline of forest ecological functions. Overgrazing and indiscriminate cultivation have caused soil sanding. Overexploitation has also caused serious erosion of black soil resources. Meanwhile, the serious brain drains in the northeast and the traditional development model based on heavy industry also hinder agricultural intensification.

### Ethics approval and consent to participate

This study involves the macro data of human economy and society. All the data are from the official statistical yearbook. The data collection process is in line with the ethical and moral standards. The research method of this study is DEA and spatial econometrics, and there is no need for ethical approval and animal experiment content. The author guarantees that the process, content and conclusion of this study do not violate the theory and moral principles.

## Empirical results

### Benchmark regression results

Before performing the spatial econometric analysis, it is necessary to empirical analyze the panel model without including spatial factors. Table 2 shows the mixed OLS regression, random effects regression, and fixed effects regression. According to the results of the F-test and the Hausman test, the random effects model is optimal.

**Table 2:** Estimation results without spatial interaction effects.

Model	AGTFP			TVA	
	1 OLS	2 RE	3 FE	4RE	5FE
AFE	− 0.050*** (− 3.30)	− 0.063*** (− 3.46)	− 0.060** (2.25)	0.354*** (4.40)	0.287*** (3.83)
Edu	0.082*** (2.94)	0.093*** (2.71)	0.102** (2.31)	0.103 (0.37)	0.177 (1.44)
Indi	0.063** (2.12)	0.072* (1.68)	0.238 (1.62)	− 2.474*** (− 6.70)	− 2.901*** (− 7.03)
Tech	0.014 (1.23)	0.013 (0.91)	0.023 (1.27)	0.415*** (7.14)	0.377*** (7.43)
Dis	0.001 (0.01)	− 0.007 (− 0.43)	− 0.028* (− 1.42)	0.038 (0.59)	0.088 (1.58)
Agri	0.024*** (3.18)	0.030*** (2.90)	0.079*** (3.17)	0.234*** (3.28)	− 0.002 (− 0.03)
Obs	390	390	390	390	390
R^2^	0.10	0.28	0.26	0.54	0.56
F test			4.07 ***		143.12***
LM test		18.09***		599.05***	
Hausman test			10.52		130.15***

*, **, *** indicate significance at the 10%, 5% and 1% level, the standard errors are in parentheses.

According to the benchmark regression results, AFE will reduce AGTFP at the 1% level, that is, a 1% increase of AFE will decrease AGTFP by 0.063%. In Model 4 and Model 5, the total output value of agriculture (TVA) is used as the dependent variable to analyze the impact of AFE on agricultural output. The results show that the expansion of government spending on agriculture can significantly increase agricultural output under the two models. It reflects that the government's policy to support agriculture will channel agricultural producers to expand production under current conditions, increase pollutant emissions and reduce AGTFP. For the control variables, Edu has a positive effect on AGTFP, in other words, every 1% increase in Edu will increase the growth of AGTFP by 0.093%, which is significant at the 1% level. The improvement of education level can improve the quality of farmers, which is conducive to the transformation of modern agriculture and promote agricultural green efficiency. Indi can also promote the growth of AGTFP, and every 1% increase in Indi will promote the growth of AGTFP by 0.093%, which is significant at the 10% level. The widening of the urban–rural income gap will cause the loss of agricultural labor force to a certain extent and reduce the amount of extensive type of input and output. At the same time, the movement of rural labor to cities can reduce the cost of urban labor and the production cost of technical tools such as agricultural machinery, thus enhancing agricultural technology to promote AGTFP. At the 1% level, a 1% increase in Agri will reduce AGTFP by 0.03%. The expansion of agricultural scale is conducive to intensive production, improving scale efficiency, and strengthening division of labor to promote AGTFP.

### Test of spatial correlation

**Figure 5:**
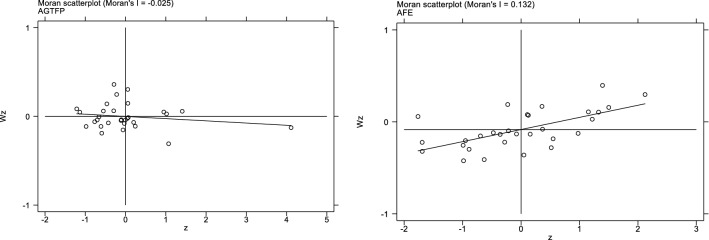
Local moran’I scatter plot based on data of 2014.

Before the spatial modeling, this paper constructs the global Moran index using the weight matrices W1 and W2 firstly. As can be seen from Table 3, there is a significant spatial positive correlation of AFE in each province. Under the W1 and W2 matrices, the AFE of each province has passed the 1% significance test. In contrast, the significance of cross-sectional data AGTFP is weaker, and there are only a few years with positive coefficients and significant spatial autocorrelation. It reflects that the spatial dependence of AGTFP in individual years is unstable. The local Moran 'I scatter plot (Fig. 5) shows the aggregation of AGTFP with AFE under the W1 matrix. Among them, the first, second, third and fourth quadrants are high-high aggregation, low–high aggregation, low-low aggregation and high-low aggregation, respectively. The local scatter of AFE is mainly located in the first and third quadrants, indicating that areas with similar agricultural financial expenditure are more likely to be clustered. And the local scatter plot of AGTFP is mainly located in the second and fourth quadrants, indicating that the spatial variability of agricultural green development is greater in that year. Meanwhile, it can be seen that AGTFP is opposite to AFE for most of the samples. The two have a negative correlation, which is consistent with the baseline regression results and provides support for the subsequent spatial analysis. However, it is worth noting that AGTFP aggregation exists instability. The spatial interaction effect of agriculture is not yet sufficient and needs further analysis and discussion.

**Table 3:** The global Maran index under W1 and W2.

Year	W1		W2	
	AGTFP	AFE	AGTFP	AFE
2008	0.005	0.130***	− 0.040	0.241***
2009	− 0.001*	0.125***	− 0.008	0.235***
2010	0.024**	0.136***	0.005**	0.235***
2011	− 0.011	0.148***	− 0.005	0.245***
2012	− 0.006	0.151***	0.013	0.242***
2013	− 0.039	0.143***	− 0.044	0.231***
2014	− 0.025	0.132***	− 0.032	0.217***
2015	0.001*	0.131***	− 0.008	0.206***
2016	0.110***	0.127***	0.052**	0.192***
2017	− 0.011	0.119***	− 0.017	0.177***
2018	0.084***	0.144***	0.060**	0.179***
2019	0.054***	0.140***	0.070**	0.181***
2020	− 0.034	0.138 ***	− 0.034	0.193***

*, **, *** indicate significance at the 10%, 5% and 1% level. For the estimated coefficients, the standard errors are in parentheses.

### Spatial econometric model

Table 4 shows the regression results of the spatial econometric models. Models 6 and 7 are based on W1 and W2 matrices respectively. First of all, the AGTFP of each province exists significant spatial correlation, and the spatial lag coefficient of AGTFP is significantly positive. It indicates that the "radiation effect" of green agriculture development in each province is significant, and it is important to strengthen the collaborative development of agriculture in each province. For every 1% increase in the local AGTFP, the AGTFP of geographically close area will increase by 0.449%, and the provinces with similar economic and geographical distance will increase by 0.355%. Secondly, the increase of AFE will suppress the local AGTFP level, supporting hypothesis 1. According to the model 7, the increase of AFE will reduce the local AGTFP by 0.037%, which is significant at the 5% level. The expansion of agricultural support expenditures has resulted in more subsidies for agricultural producers in the region. As farmers expand production, agricultural pollution emissions increase, thus reducing the AGTFP in the local area. With the W2 matrix, the AFE in this region has a more significant effect on the AGTFP in this region. In addition, the − 0.025 in model 6 is not statistically significant, but it still has economic implications. The coefficient indicates the impact of AFE on AGTFP within the region. The model assumes that this coefficient is not driven by the surrounding area. Therefore, it is equally reasonable to use the economic geographic nested matrix and the geographic distance matrix. Obviously, AFE can significantly reduce AGTFP in the surrounding area. An increase of 1% in the local area of AFE will lead to a decrease of about 0.123% and 0.116% in geographically similar areas and economically geographically similar areas of AGTFP respectively. Therefore, Hypothesis 2 is supported. With the increase in AFE in this region, the agricultural productivity and agricultural efficiency will improve in this region, which has a "demonstration effect" on the surrounding areas. It is obvious that the surrounding provinces will expand their production and reduce the AGTFP.

**Table 4:** Regression results of AFE on AGTFP.

	6 SDM (W1)	7 SDM (W2)
W*AGTFP	0.449*** (4.62)	0.355*** (3.76)
AFE	− 0.025 (− 1.30)	− 0.037** (− 1.93)
Edu	0.062* (1.83)	0.060* (1.70)
Indi	0.030 (0.59)	0.020 (0.38)
Tech	0.020 (1.40)	0.013 (0.86)
Dis	− 0.015 (− 0.93)	− 0.012 (− 0.76)
Agri	0.028*** (2.19)	0.027*** (2.60)
W*AFE	− 0.123** (− 2.04)	− 0.116** (− 2.28)
W*Edu	0.129 (1.52)	0.145* (1.77)
W*Indi	0.240 (1.54)	0.246 (1.61)
W*Tech	− 0.011 (− 0.24)	0.050 (1.06)
W*Dis	− 0.048 (− 1.06)	− 0.060 (− 1.43)
W*Agri	− 0.071 (− 1.58)	0.010 (0.23)
Obs	390	390
R^2^	0.39	0.36
Log-likelihood	477.460	473.534
Hausman test	34.74 ***	39.91***

*, **, *** indicate significance at the 10%, 5% and 1% level. For the estimated coefficients, the standard errors are in parentheses.

Table 5 further decomposes the direct effects, indirect effects of models 6 and 7. The results show that the reduction effect of AFE on AGTFP in the surrounding areas is extremely significant regardless of the use of W1 and W2. For every 1% increase of AFE in this province, the AGTFP in geographically similar areas and economically geographically similar areas will decrease by about 0.247% and 0.202% respectively. Agricultural producers in similar provinces are vulnerable to the "demonstration effect". The increase in agricultural support in the local area will lead to better psychological expectations and higher production motivation of agricultural producers in the surrounding provinces. In addition, the direction of coefficient for control variable is basically the same. The direct and indirect effects of Edu on AGTFP are both positive, which further reflecting the importance of education for green agricultural development. In general, Indi will increase the growth of AGTFP. The large widening of urban–rural income gap will undoubtedly lead to agricultural labor outflow. On one hand, it will reduce the current production scale. On the other hand, it will reduce the cost of urban labor and increase the utilization rate by reducing the cost of agricultural machinery and fertilizer, thus enhancing agricultural technology and improving AGTFP. Agri can enhance the AGTFP in the local area, reflecting the contribution of intensive production to agricultural development.

**Table 5:** Direct, indirect, and total effects under SDM.

	Model-6			Model-7		
	Direct	Indirect	Total	Direct	Indirect	Total
AFE	− 0.030 (− 1.52)	− 0.247** (− 2.44)	− 0.276*** (− 2.80)	− 0.040** (− 2.06)	− 0.202*** (− 2.75)	− 0.242*** (− 2.75)
Edu	0.066* (1.95)	0.292** (2.03)	0.358** (2.47)	0.063* (1.79)	0.265** (2.26)	0.265*** (2.26)
Indi	0.045 (0.96)	0.456 (1.60)	0.501* (1.82)	0.033 (0.68)	0.387 (1.64)	0.387* (1.64)
Tech	0.020 (1.47)	− 0.010 (− 0.12)	0.010 (0.12)	0.014 (1.01)	0.080 (1.12)	0.080 (1.12)
Dis	− 0.017 (− 1.09)	− 0.097 (− 1.55)	− 0.114 (− 1.31)	− 0.014 (− 0.89)	− 0.097 (− 1.47)	− 0.097 (− 1.47)
Agri	0.026*** (2.78)	− 0.117 (− 1.38)	− 0.090 (− 1.07)	0.027*** (2.77)	0.022 (0.33)	0.022 (0.33)

*, **, *** indicate significance at the 10%, 5% and 1% level. For the estimated coefficients, the standard errors are in parentheses.

### Regression results in different periods

**Figure 6:**
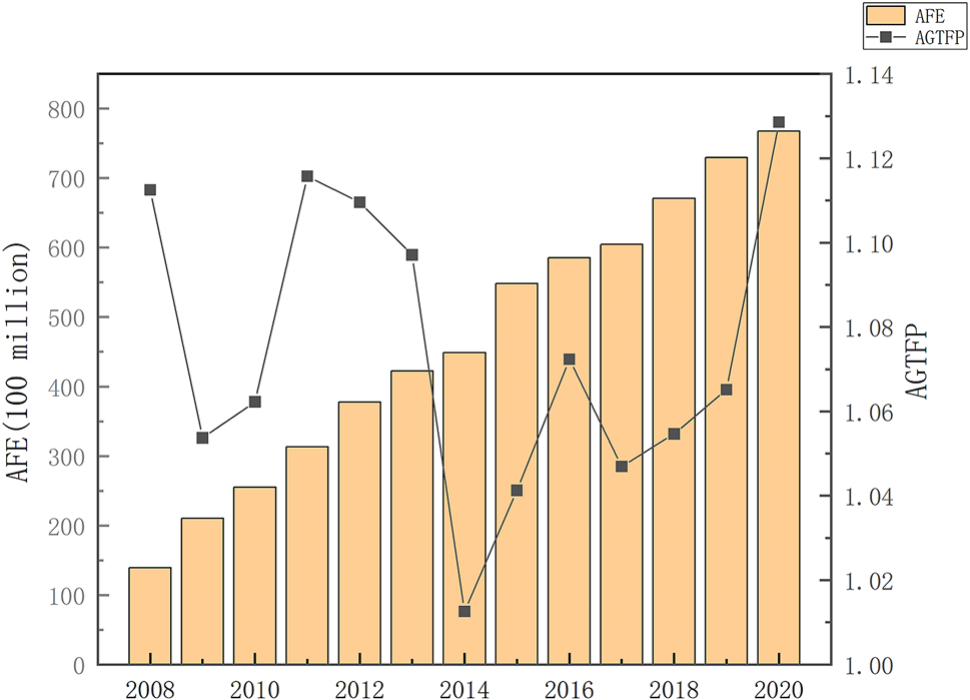
Characteristics of AGTFP and AEF temporal changes from 2008 to 2020.

Figure 6 shows the changes of the original AGTFP and AFE in 30 provinces over 13 years, reflecting the original time-varying characteristics of both. AGTFP has increased year after year and basically remained between 1.00 and 1.10. In particular, the financial crisis in 2008 directly affected some agricultural exports and reduced the export trade turnover. It should note that the financial crisis caused a plunge in agricultural prices, thus resulting in a drop of AGTFP in 2009. The "Three Rural Policies" issued in 2004 have deepened the rural reform, improved and strengthened the policy to support farmers, and stabilized the overall development of the rural economy. Therefore, from 2009 to 2011, AGTFP always maintained the growth trend. While from 2011 to 2014, with the impact of the financial crisis gradually weakened, the secondary and tertiary industries grew rapidly and the modernization process accelerated. However, there was a lack of agricultural modernization, and AGTFP dropped sharply. In 2015, the first central document officially proposed to build modern agriculture, accelerate the transformation of agricultural development, and promote the modernization of agriculture. It includes focusing on innovation in agricultural science and technology and strengthening the driving role of agricultural science and technology. Obviously, this policy has transformed the crude mode of operation that relies on resource consumption in agriculture, improved the content of agricultural science and technology, and achieved successive increases in AGTFP. The AFE has increased in successive years during the sample period, especially in 2009. The No. 1 Central Document of 2009 emphasized further increasing agricultural input, substantially increasing agricultural subsidies, and strengthening policies that benefit farmers. However, the AGTFP did not increase accordingly, reflecting the underutilization of AFE in the early years. The increase in farm support expenditures, on the contrary, exacerbated agricultural pollution. In the later period, with AFE increased in successive years, the negative effect of AGTFP and AFE is weaken.

According to the above analysis, AFE can significantly reduce AGTFP. It is worth noting that in 2015, the No. 1 Central document officially proposed that the construction of modern agriculture should focus on reform and innovation. To further analyze the impact of policies on AGTFP, 2015 is used as the cut-off point for time heterogeneity analysis. Table 6 shows that the AGTFP has significant positive spatial spillovers under both W1 and W2 before the promulgation of the document, that is, a 1% increase of AGTFP in the local area will increase AGTFP in the surrounding geographically similar provinces by about 0.437%; and an increase of 0.400% in the surrounding economically geographically similar provinces, both significant at the 1% level. In addition, the spatial effect of AGTFP is not significant after 2015. It indicates that the linkage spillover effect of provinces is obvious in the early stage of agricultural modernization construction, and there are significant technology spillover and configuration spillover. Along with the modernization and transformation, the provinces are more inclined to develop characteristic agriculture according to local conditions by taking into account the actual situation of the region. At the same time, in order to further develop modern agriculture in the region, the competitive pressure of each province has increased, thus strengthening the "siphon effect". Provinces increase the plunder of human capital and other factors of production, resulting in the spatial spillover of AGTFP decreases. According to model 8 and model 9, AFE can significantly affect AGTFP in the surrounding areas, an increase of 1% of AFE in the local area will decrease the AGTFP in geographically similar areas and economically geographically similar areas by about 0.219% and 0.179%, which is significant at the 1% level. The possible reason is that with the advanced of modernization, agricultural producers in the surrounding provinces tend to shift from rough production to intensive agriculture. Therefore, AFE does not inhibit the increase of AGTFP after 2015.

**Table 6:** Time classification regression.

SDM	2008–2014		2015–2020	
	8	9	10	11
	W1	W2	W1	W2
W*AGTFP	0.437*** (3.34)	0.400*** (3.22)	0.150 (0.71)	0.036 (0.20)
AFE	− 0.005 (− 0.25)	− 0.002 (− 0.09)	− 0.036 (− 1.26)	− 0.045 (− 1.48)
Edu	0.038 (1.21)	0.026 (0.74)	0.069 (1.26)	0.085 (1.44)
Indi	0.074* (1.87)	0.054 (1.27)	− 0.017 (− 0.24)	0.005 (0.07)
Tech	− 0.013 (− 0.84)	− 0.017 (− 1.05)	0.029 (1.32)	0.015 (0.63)
Dis	− 0.020 (− 1.40)	− 0.019 (− 1.25)	0.019 (0.41)	− 0.007 (− 0.16)
Agri	–0.003 (− 0.24)	–0.006 (− 0.53)	0.027 (2.11)	0.023 (1.63)
W*AFE	− 0.219*** (− 3.24)	− 0.179*** (− 3.01)	− 0.008 (− 0.04)	0.178 (1.11)
W*Edu	0.268*** (2.76)	0.261*** (2.85)	− 0.022 (− 0.06)	− 0.369 (− 1.29)
W*Indi	− 0.178 (− 1.33)	− 0.036 (− 0.28)	0.854** (2.13)	0.525 (1.31)
W*Tech	− 0.101 (− 1.58)	− 0.018 (− 0.29)	0.067 (0.68)	0.168** (2.01)
W*Dis	− 0.056 (− 1.33)	− 0.046 (− 1.13)	0.156 (0.44)	− 0.207 (− 1.02)
W*Agri	− 0.016 (− 0.34)	0.043 (0.89)	− 0.105 (− 1.18)	0.032 (0.39)
Obs	390	390	390	390
R^2^	0.40	0.22	0.56	0.40
Log-likelihood	298.784	292.588	200.523	200.193
Hausman test	98.55***	75.16***	38.29***	23.39***

*, **, *** indicate significance at the 10%, 5% and 1% level. For the estimated coefficients, the standard errors are in parentheses.

Finally, Table 7 reports further analysis of Models 8 and 9, and the conclusions are generally consistent with the previous results. AFE can reduce AGTFP in surrounding areas, and the negative spatial spillover effect is significant. It indicates that the counteracting effect of extensive agriculture to policy in the early stage of modernization construction. In terms of control variables. A 1% increase in Edu will increase AGTFP by about 0.599% in geographically similar provinces and 0.458% in economically geographically similar provinces. The increase in education level has a significant impact on green progress in peripheral agriculture. Indi will significantly improve AGTFP in this region. A 1% increase in Indi in the local area will increase AGTFP in the local area by 0.073%. The loss of agricultural personnel caused by the widening gap between urban and rural areas in this province will drive the improvement of agricultural efficiency by reducing the labor cost of urban agricultural science and technology products in the short term. Tech has a negative spillover for AGTFP in geographic proximity. It indicates that in the early stage of agricultural modernization, the investment in science and technology has insufficient positive influence on the surrounding areas. It also reflects the lack of innovation awareness of producers in the early stage, and the "learning effect" of local innovation activities is not obvious, so they blindly increase output and expand extensive output.

**Table 7:** Direct, indirect, and total effects under Model-8 and Model-9.

	Model-8			Model-9		
	Direct	Indirect	Total	Direct	Indirect	Total
AFE	− 0.002 (− 0.11)	− 0.393*** (− 3.13)	− 0.395*** (− 3.13)	− 0.040 (− 2.06)	− 0.301*** (− 3.29)	− 0.309*** (− 3.31)
Edu	0.046 (1.47)	0.559*** (3.21)	0.559*** (3.41)	0.063 (1.79)	0.458*** (3.37)	0.491*** (3.59)
Indi	0.073** (2.02)	− 0.266 (− 0.98)	− 0.193 (− 0.72)	0.033 (0.68)	− 0.027 (− 0.12)	0.031* (0.15)
Tech	− 0.017 (− 1.12)	− 0.194* (− 1.73)	− 0.210* (− 1.88)	0.014 (1.01)	− 0.044 (− 0.44)	− 0.061 (− 0.61)
Dis	− 0.022 (− 1.64)	− 0.116 (− 1.33)	− 0.139 (− 1.54)	− 0.014 (− 0.89)	− 0.088 (− 1.23)	− 0.109 (− 1.48)
Agri	− 0.004 (− 0.34)	− 0.043 (− 0.48)	− 0.047 (− 0.53)	0.027 (2.77)	0.061 (0.74)	0.056 (0.68)

*, **, *** indicate significance at the 10%, 5% and 1% level. For the estimated coefficients, the standard errors are in parentheses.

### Robustness test

There have been various measures of total factor productivity, and to further analyze the model robustness, this paper uses the Directional Distance Function (DDF) to re-measure the AGTFP as the dependent variable. The Directional Distance Function model (DDF) is a generalized expression of the radial DEA model and is able to differentiate between desirable and undesirable outputs. In which, the directional distance function is set as: ${D}_{0}=\left(x,y,b:{g}_{y},-{g}_{b}\right)=sup\left\{\left(\beta :y+\beta {g}_{y},b-\beta {g}_{b}\right)\epsilon S\left(x\right)\right\}$, $\beta$ is the degree of expansion of agricultural output along the directional vector ${g}_{y},$ and the degree of contraction of agricultural pollution emissions along the directional vector $-{g}_{b}$. The ratio of agricultural fiscal expenditure/total fiscal expenditure is also used as the core explanatory variable to jointly measure the robustness of the model.

The regression results are shown in Table 8, and the results indicate that the values of these coefficients do not change significantly and there is no change in direction and significance. The empirical results of this paper are robust.

**Table 8:** Robustness test.

SDM	W1	W2
W*AGTFP (DDF)	0.387*** (3.77)	0.230*** (2.26)
AFE	− 0.003 (− 0.20)	− 0.009 (3.24)
W*AFE	− 0.095* (− 1.79)	− 0.114** (− 2.52)
Control variables	Yes	Yes

*, **, *** indicate significance at the 10%, 5% and 1% level. For the estimated coefficients, the standard errors are in parentheses.

### Endogeneity test

Since policy, institutional and other factors may exist in residuals, it may result in a problem of omitted variable. Therefore, this paper conducts endogeneity test, adopts instrumental variable IV estimation to conduct two-stage least squares analysis. The instrumental variables should satisfy the assumptions of "independent of the disturbance term" and "correlated to endogenous variables". Referring to Zhong et al.^51^, the sample period (2003–2015) of mailboxes number has a five-year time lag with the empirical sample. The instrumental variable has a strong historical attribute. So the disturbance term in the sample period cannot affect the instrumental variables, which satisfies the independence condition. Therefore, the number of mailboxes (MN) is used as the instrumental variable, and the column (1) in Table 9 shows the regression results between the instrumental variable and AFE. The regression coefficient of the two is -0.084, which is significant at 1% level, indicating that MN has a significant negative impact on AFE. Column (2) reports the results of the two-stage IV regression. The AFE regression coefficient is − 0.109, which is significant at the 1% level, and the results are basically consistent with the benchmark regression. In addition, considering the possible weak correlation between instrumental variables and endogenous variables, the lag period of independent variable is added as another instrumental variable. Similar to the above instrumental variables, this instrumental variable also has a certain historical attribute. The regression results of both instrumental variables show a significant negative effect of AFE on AGTFP. In summary, the robustness of the basic conclusion has been further verified after considering the endogeneity problem.

**Table 9:** Regression results of instrumental variable.

	OLS (AFE)	SLS (AGTFP)	OLS (AFE)	SLS (AGTFP)
AFE		− 0.109*** (− 2.90)		− 0.035* (− 1.94)
IV:MN	− 0.084*** (− 8.60)			
IV: L.X1			0.812*** (34.45)	
Edu	1.303*** (23.21)	0.175*** (2.89)	0.303*** (7.03)	0.073** (2.33)
Indi	− 0.206** (− 2.19)	0.040 (1.20)	− 0.123** (− 2.42)	0.049 (1.53)
Tech	− 0.171*** (− 4.75)	0.007 (0.54)	− 0.068*** (− 1.62)	0.019 (1.63)
Dis	0.119*** (2.71)	0.006 (0.38)	0.032 (1.32)	− 0.008 (− 0.55)
Agri	0.162*** (7.42)	0.033*** (3.56)	0.022* (1.83)	0.022*** (2.84)
Obs	390	390	390	390
R^2^	0.86	0.06	0.96	0.10
Hausman test		16,095.02***		79,494.57***

*, **, *** indicate significance at the 10%, 5% and 1% level. For the estimated coefficients, the standard errors are in parentheses.

### Discussion

According to the empirical results above, the main findings are as follows:

Firstly, AGTFP has a significant positive spatial spillover effect. The spatial coefficients of AGTFP are positive under both matrices. The main reason is that close areas often have similar geographical environment, economic characteristics and natural resources. The cost of interconversion of agricultural production factors in similar provinces is low. For example, Zhou et al.^52^ believed that agricultural soil and water resources in neighboring areas can have a positive impact on mutual agricultural economy, and the cost of mutual transformation of agricultural production factors in similar provinces is low. Meanwhile, production factors such as knowledge and technology are especially characterized by high mobility, and the interactive flow of production factors can significantly affect AGTFP in surrounding areas. This conclusion has been confirmed by scholars. Yang et al.^53^ proposed that as agricultural mechanization has become one of the main reasons for the continuous growth of agricultural production in China, labor transfer and local non-farm employment may enable farmers to use higher quality grain production inputs, thus promoting agricultural economic development. Varshney et al.^54^ also found that spillover of agricultural frontier technology can significantly improve the level of agricultural development. In addition, the agricultural production activity itself exists geographical correlation. In particular, the process of intensive agricultural development has been accelerated, and the specialization of agricultural production has become clearer. With the increase of inter-provincial agricultural cooperation, AGTFP spillover effect is improved.

Secondly, the increase of AFE will inhibit the level of AGTFP. AFE is the main channel and policy tool of the government to support agriculture. Krmpot and Gajdobranski^55^ analyzed the agricultural development of Serbia and found that a large amount of financial investment in agriculture could make food supply stable. Agricultural producers can expand their production scale with the help of financial support. The original intention of the increase in AFE is that the government aims to improve the scientific and technological content of agriculture, change the mode of agricultural development and improve green production. In October 2008, the Third Plenary session of the 17th CPC Central Committee formally put forward the agricultural modernization strategy and formulated a series of long-term development plans, in order to promote agricultural development and agricultural mechanization level, and achieve the goal of agricultural modernization. However, in the early stage of modernization, the agricultural production mode is low. Zhang et al.^56^ proposed that irrigation methods in China's agriculture are inefficient and wasteful, and there is ample room for the development of agricultural science and technology. Agricultural producer still has extensive model development consciousness. There is excessive use of synthetic nitrogen fertilizers^57,58^, causing significant losses and serious environmental externalities^59^. Farmers use the support funds to expand production scale, increase the output and discharge more pollution, thereby reducing AGTFP in the region. For the surrounding provinces, the provinces are sensitive to the agricultural production conditions in the surrounding areas, and the "learning effect" is significant. On one hand, with the improvement of AFE in this region, infrastructure is improved, agricultural subsidies are increased, and the level of agricultural mechanization is improved as well. Obviously, this will produce "demonstration effect" to the surrounding province, but also enhance the confidence of the production and operation of the surrounding province. On the other hand, the surrounding agricultural producers find that the local government increases financial support for agriculture, and their expectations are positive, believing that the surrounding governments will also increase their efforts to benefit agriculture. Thus, the initiative to produce increases. But at present, the power of agricultural transformation is insufficient, and it is difficult to complete the transformation from extensive production mode to intensive production mode by itself. The only way to increase output is to expand reproduction, thereby reducing the surrounding AGTFP.

Finally, considering the time heterogeneity, the inhibitory effect of AFE on AGTFP is particularly significant before 2015. In 2015, the government proposed to intensify reform and innovation and accelerate rural modernization. On the basis of the previous agricultural development strategy, the government puts forward to focus on innovation and reform, thus changing the way agricultural producers use AFE. Ragasa and Babu^60^ proposed that appropriate policy reforms are needed to achieve food security for all in developing countries. Gong^61^ used variable coefficient production function to capture the structural changes of six reform and innovation periods in the past 40 years, and found that the production process and technology of the four agricultural industries (agriculture, forestry, animal husbandry and fishing) were different, and agricultural technology and input could alternately lead economic growth in different reform periods. From 2008 to 2014, residents' awareness of environmental protection is weak, and financial support for agriculture is more used to increase output. At this time, the agricultural economy relies more on the increase of input factors. There is insufficient awareness of changing the industrial mode. Therefore, in the early stage of agricultural modernization strategy, the increase of agricultural financial support will inhibit AGTFP in neighboring provinces. With the introduction of the concept of green development, the government's emphasis on agricultural transformation has deepened. From the current crude agriculture with low technological content and primitive farming methods, it gradually turns into intensive agriculture with high mechanization and advanced agricultural technology. At this time, the use of AFE by agricultural producers shifted to improving the production model and increasing the degree of mechanization. Therefore, after the reform and innovation are proposed, AFE no longer has a suppressive effect on AGTFP in neighboring provinces.

## Conclusion and policy recommendations

The main conclusions are as follows: (1) AGTFP has a significant positive spatial spillover effect. The "radiation effect" of agricultural green development is significant. (2) AFE can significantly reduce AGTFP in the local area. A 1% increase of AFE in the local area will increase AGTFP in the local area by 0.037%. The increase of financial assistance to agriculture in the region drives the scale of local agricultural production, which increases pollution emissions. (3) AFE has a significant negative spatial spillover on AGTFP. A 1% increase of AFE in the local area will increase AGTFP by about 0.123% in geographically similar provinces and 0.116% in economically geographically similar provinces. The AFE leads to the optimization of agricultural production conditions in the province and has a "demonstration effect" on the surrounding areas. (4) According to the analysis of different periods, AFE has a negative impact on AGTFP mainly before the reform and innovation is proposed in 2015. It indicates that the policy promulgation has a significant impact on agricultural emission reduction. Based on theoretical analysis and empirical conclusions, this study puts forward the following policy recommendations.

1. First, the green development of provincial agriculture needs to coordinate regional agriculture. Agricultural production is more dependent on natural environment and natural resources, and it has obvious characteristics of regional linkage. In the future, the coordinated development of regional agricultural green transformation should be strengthened. The government should play the role of demonstration and leadership of the strong green development province to the surrounding area, and strengthen the exchange and learning of advanced technology and management experience. It should promote regional agricultural transformation through regional agricultural technology innovation. (2) Secondly, the government needs to guide enterprises to operate moderately and promote agriculture from increasing production-oriented to quality-oriented. AFE is a direct channel for the government to support agriculture. It is necessary to change the obstacle to AGTFP caused by the increase of AFE, the government should moderately guide operation in the future and shift from increasing agricultural production to improving agricultural quality, that is, the government should accelerate the transformation of the agricultural development mode from extensive agriculture to intensive agriculture. Specifically, provide vocational quality training for agricultural operators and improve the utilization of financial resources; increase publicity for environmental protection and ecologically balanced agriculture, so as to promote the development of building environment-friendly agriculture; efficient use of infrastructure improvements brought about by the increase in AFE. At the same time, the government should also focus on land quality, subsidize producers who introduce pollution treatment equipment, and strictly prevent and control pollution. (3) Finally, the agricultural sector needs to increase scientific research and develop biological agriculture to improve agricultural science and technology content. After proposing innovative agriculture, AFE no longer inhibits green progress in agriculture. It further illustrates that innovation can greatly improve the efficiency of agricultural producers' use of agricultural support funds. In the future, the sector should upgrade the level of mechanization and pay attention to updating advanced equipment. The producer should also strengthen the role of innovation in promoting agriculture, screen and grasp the best varieties, and the government should provide ecological compensation to agricultural producers who make technological innovations and production improvements. In addition, it is necessary to promote green and efficient pesticides on a large scale, improve the regulatory system of agricultural film recycling, and build livestock and poultry manure treatment equipment to reduce agricultural non-point source pollution emissions. At the same time, through the development of biological agriculture to promote the formation of circular bioeconomy. Leading sustainable development with circular bioeconomy. We should make use of our abundant germplasm resources to develop biological breeding, and actively promote the breeding of high-quality grain crops. We need to carry out scientific breeding of high-quality pigs, dairy cows, broilers and other livestock and aquatic products. Meanwhile, it is necessary to develop synthetic biology technologies to promote the substitution of biobased products for synthetic fertilizers, pesticides and agricultural plastics. In addition, biotechnology should be used to improve China's marginal land resources and promote the restoration of land quality. Thus, the production capacity and quality of agriculture are improved and the environmental pressure of agricultural development is reduced.

## Supplementary Information

Supplementary Tables.

## Data Availability

The datasets generated during and/or analysed during the current study are available from the corresponding author on reasonable request.
