# Supplementary material for: The inhibitory effect of agricultural fiscal expenditure on agricultural green total factor productivity
Source: Sci Rep. 2022 Dec 3;12:20933. doi: 10.1038/s41598-022-24225-2 (PMC9719522; doi:10.1038/s41598-022-24225-2)
Supplement: Supplementary file 1 — Supplementary Tables. [file 41598_2022_24225_MOESM1_ESM.docx]

**The inhibitory effect of agricultural fiscal expenditure on agricultural green total factor productivity**

First author:Shuguang Wang^1^

Second author:Jiaying Zhu^2^

Third author:Lang Wang ^3^

Fourth author:Shen Zhong ^4&*^( Corresponding author)

^1，2,4&*^ Harbin University of Commerce, Harbin, Heilongjiang, China

^3^School of Finance, Dongbei University of Finance and Economics, Dalian, Liaoning, China

E-mail: 102159@hrbcu.edu.cn (SG.Wang)

E-mail: zjy@s.hrbcu.edu.cn (JY.Zhu)

E-mail: wanglang@hrbcu.edu.cn (L.Wang)

E-mail: 102714@hrbcu.edu.cn (S.Zhong)

**Supplementary Table**

Table S1 Variables under the entropy method

| Variable | Entropy value ($e_{j}$) | Differentiation factor ($g_{j}$) | Weight ($w_{j}$) |
| --- | --- | --- | --- |
| COD | 0.8950 | 0.1050 | 0.3171 |
| AN | 0.9079 | 0.0921 | 0.2781 |
| TN | 0.9455 | 0.0545 | 0.1645 |
| TP | 0.9205 | 0.0795 | 0.2402 |

Table S2 Panel Unit Root Test

| Variable | Period | Panel | HT test |
| --- | --- | --- | --- |
| AGTFP | 15 | 30 | 0.020*** |
| AFE | 15 | 30 | 0.386** |
| Edu | 15 | 30 | 0.390** |
| Indi | 15 | 30 | -0.210*** |
| Tech | 15 | 30 | 0.404** |
| Dis | 15 | 30 | -0.120*** |
| Agri | 15 | 30 | 0.421* |

Notes: *, **, *** indicate significance at the 10%, 5% and 1% level, the standard errors are in parentheses.
